# A positive effect of flowers rather than eye images in a large-scale, cross-cultural dictator game

**DOI:** 10.1098/rspb.2012.0758

**Published:** 2012-06-06

**Authors:** Nichola J. Raihani, Redouan Bshary

**Affiliations:** 1 Department of Genetics, Evolution and Environment, University College London, Gower Street, London WC1E 6BT, UK; 2 Department of Biology, University of Neuchâtel, Emile-Argand 11, Neuchâtel 2009, Switzerland

**Keywords:** dictator game, human cooperation, eye images, fairness, reputation, cooperative behaviour

## Abstract

People often consider how their behaviour will be viewed by others, and may cooperate to avoid gaining a bad reputation. Sensitivity to reputation may be elicited by subtle social cues of being watched: previous studies have shown that people behave more cooperatively when they see images of eyes rather than control images. Here, we tested whether eye images enhance cooperation in a dictator game, using the online labour market Amazon Mechanical Turk (AMT). In contrast to our predictions and the results of most previous studies, dictators gave away more money when they saw images of flowers rather than eye images. Donations in response to eye images were not significantly different to donations under control treatments. Dictator donations varied significantly across cultures but there was no systematic variation in responses to different image types across cultures. Unlike most previous studies, players interacting via AMT may feel truly anonymous when making decisions and, as such, may not respond to subtle social cues of being watched. Nevertheless, dictators gave away similar amounts as in previous studies, so anonymity did not erase helpfulness. We suggest that eye images might only promote cooperative behaviour in relatively public settings and that people may ignore these cues when they know their behaviour is truly anonymous.

## 1. Introduction

Humans rarely behave according to economic theories of self-interest. A slew of real-world and laboratory experiments have shown that, while taking their own material payoffs into account when making decisions about resource allocation in social interactions, humans are also sensitive to social norms, fairness preferences and how others might respond to their behaviour. One striking result is that people behave more cooperatively when observed or when their decisions will be made known to others than when decisions are made anonymously [1–8]. Investment in reputation is beneficial because cooperative individuals may be more likely to receive help from others in the future [1,2,9], or because it increases the chance that they will be chosen for cooperative partnerships [6,8,10,11]. Recent empirical work has suggested that concerns about reputation can be activated via minimal social cues of being watched, such as artificial eye images [12–17]. However, there is still considerable debate concerning the conditions under which such cues produce measurable effects [18–21]. To date, no study has examined whether there is cross-cultural variation in the effects of eye images on subject behaviour in economic games. This is an oversight because it is now acknowledged that the typically used Western, educated, industrialized, rich and democratic (WEIRD) undergraduate subjects are unrepresentative of the human race as a whole [22]. Indeed, studies conducted using a more diverse subject pool have often found striking differences in social behaviour when cultural differences are taken into account [23–26].

A simple game to investigate human decision-making in a social context is the dictator game [27,28]. In this two-player game, a dictator is endowed with a sum of money, which he can choose to split with a receiver. The receiver in this game is powerless and must accept any division of the endowment proposed by the dictator. Economic models based on narrow self-interest predict that dictators should keep the entire endowment, leaving receivers with no share. However, this prediction has been universally rebutted by empirical data (see [28,29]). Instead, people are typically far more altruistic than would be expected, with mean allocations to receivers averaging around 28 per cent [29]. Rather than continuing to emphasize that empirical data falsify the predictions of the *Homo economicus* model of human behaviour, attention has now turned to explaining the considerable heterogeneity in allocations in the dictator game (see Engel [29] for a meta-analysis). In particular, dictators appear to be sensitive to their reputation: dictators tend to make lower allocations to receivers when their anonymity is protected [28]. In addition, reducing the social distance between dictators and receivers by identifying or providing information about receivers can increase allocations in this game [30–32].

It is less clear whether subtle social cues, such as eye images, also serve to increase dictator generosity. It is possible that eye images activate concerns about being watched, either by other individuals or by the experimenter (if subjects are aware that they are in an experiment). In each case, we would expect eye images to have a positive effect on cooperative behaviour. Eye images have been shown to increase dictator donations to receivers in laboratory games [12,15,17] and subsequent studies have also demonstrated that eye images can increase prosocial behaviour in other contexts [13,14,16]. However, these results are not universally supported. In a game where players were entrusted with an endowment from another player, Fehr & Schneider [18] found no effect of eye images on the propensity of the trustees to altruistically send the money back to the truster. A recent study failed to find any positive effect of eye images on cooperative behaviour [19], while others have shown that subtle eye cues may only increase cooperative behaviour when there are few potential observers present [20]. Recent experiments under field and laboratory settings have also demonstrated that people recognize when their social decisions are truly anonymous and that they may not therefore respond to subtle cues of being watched [33,34]. For example, in a real-world study measuring people's donations to a church collection fund, Soetevent [33] showed that people donated less when the collections were placed in closed bags rather than open (and therefore observable) baskets. This effect persisted despite the fact that donations were made in the presence of other churchgoers who might be expected to activate subconscious concerns about reputation in the same way as has been proposed for eye images.

For the time being at least, the extent to which human social behaviour is affected by subtle social cues remains unclear. Moreover, the possibility for cross-cultural variation in responses to such cues has never been investigated. Here, we used a novel approach to test the effect of eye images on donations in the dictator game, while controlling for additional variables such as culture, age, income, education level and gender. Using the online labour market Amazon Mechanical Turk (AMT), we recruited participants from all over the world to take part in our study. Online labour markets use the Internet to connect the so-called ‘requesters’ (or experimenters) with ‘workers’ (or subjects) for small tasks, while facilitating the payment of workers for their time [35–37]. Using AMT, our subjects played a dictator game under completely anonymous, double-blind conditions.

## 2. Methods

### (a) Amazon Mechanical Turk and the dictator game

Experiments were conducted in December 2011 and March 2012 using AMT (www.mturk.com). We recruited 776 subjects, who were randomly allocated to the role of ‘decider’ (*n* = 388) or ‘receiver’ (*n* = 388). Regardless of the role they were allocated, subjects were only allowed to play the game once. Subject identities were protected since under the AMT framework both employers and workers interact using unique identity codes. Workers are prevented from accruing multiple worker IDs because each ID must be linked with a unique credit card number [37]. All subjects were informed that their worker ID would remain secret and that they would not be told the worker ID of their partner. Subjects were first asked to complete a questionnaire to obtain information about gender, age, average earnings, level of education, country of origin and current location. Subjects answer questions like these with around 97 per cent accuracy [36]. Following the questionnaire, we presented subjects with written instructions for the dictator game in English, as well as informing subjects whether they were allocated the role of dictator or receiver. For the sake of neutrality, we used the term ‘decider’ rather than ‘dictator’ and framed the task as a game involving decision-making rather than labelling it as the ‘dictator game’ (see appendix for game instructions and questionnaire). All subjects had to answer two comprehension questions correctly to ensure they understood the rules of the game. If they answered the two questions correctly, they received a $0.20 payment for taking part, regardless of their role in the game. Dictators were then told they had been allocated an additional $0.50, any amount of which they could choose to share with an anonymous receiver. Dictators were required to fill out the amount they would keep and the amount they would donate to a receiver in separate boxes. As a further test that dictators understood and were attending to the task, it was specified that the amounts in the two boxes must sum to $0.50. Dictators that did not fulfil this requirement were excluded from the experiment. Dictators in this experiment were subject to one of four treatments. Above their decision boxes, dictators were shown either an image of eyes (*n* = 92) or an image of flowers (*n* = 97) [13,16]. Fifty unique images of human eyes and flowers, respectively, were used in this study to avoid pseudo-replication. The other subjects in this study were shown a control image of a black square (*n* = 99) or no image (*n* = 100).

There are several preconceptions surrounding the use of subjects recruited from the Internet for psychological and behavioural studies. These include the idea that Internet samples represent a less diverse slice of society than those used in traditional laboratory settings and that Internet-based findings may therefore differ systematically from those obtained with other methods. However, there is little evidence to support the idea that Internet-recruited samples are less diverse than samples recruited using more traditional methods, and studies performed on the Internet have produced findings that are consistent with those obtained from other methods [38]. Previous work has shown that while the sex ratio is biased towards men, AMT workers are more demographically diverse than standard Internet samples and significantly more diverse than samples using Western undergraduates [39], and, importantly, that AMT workers are not demographic outliers with respect to other samples of the community from which they are recruited [40,41]. Moreover, behavioural experiments using AMT have also reliably replicated findings obtained using real-world laboratory methods [37,42,43].

### (b) Subject pool

Our subject pool of dictators was made up of 146 females and 240 males (two subjects did not specify gender) ranging in age from 18 to 67 years old (mean = 27 years old). All subjects had a history of completing tasks to earn money on AMT. Annual self-reported income varied from less than $12 500 to over $100 000, and education level varied widely, from high school only up to postgraduate degree level (see electronic supplementary material, table S1). Dictators were recruited from 46 countries and were grouped into ten categories, representing nine cultures according to the Inglehart & Baker [44] scheme, plus an additional category for those countries not assigned a world culture (table 1). This scheme classifies countries into distinct cultures according to historical, religious, political or value differences and has previously been used to classify participants in economic games according to culture [26]. Each of our nine cultures was represented by a minimum of two (range = 2–170) independent samples. One player did not indicate which country they were from. Six of our dictators came from countries that were not included in the Inglehart & Baker [44] scheme (table 1). Self-reported income level was significantly predicted by culture, with players from English-speaking countries reporting higher income levels than average, while players from Orthodox and South Asian countries reported lower income levels than average (results based on ordinal linear regression). There was no significant relationship between self-reported income and age or education level.

**Table 1.** Self-reported country of origin of dictators and the world culture each country corresponds to according to Inglehart & Baker [44]. Sample sizes for each country and world culture are indicated in parentheses.

world culture (*n*)	country (*n*)
Africa (4)	Ghana (1)
	Kenya (1)
	Nigeria (1)
	South Africa (1)
Catholic Europe (18)	Austria (2)
	Belgium (1)
	Croatia (2)
	Italy (3)
	Poland (3)
	Portugal (2)
	Slovakia (1)
	Slovenia (2)
	Spain (2)
Confucian (3)	China (3)
English-speaking (156)	Australia (3)
	Canada (8)
	Ireland (1)
	UK (7)
	USA (137)
Ex-communist (2)	Lithuania (2)
Latin America (7)	Argentina (2)
	Brazil (2)
	Colombia (2)
	El Salvador (1)
Orthodox (15)	Bosnia and Herzegovina (2)
	Bulgaria (1)
	Macedonia (2)
	Romania (8)
	Russia (2)
Protestant Europe (6)	Germany (1)
	Sweden (1)
	Switzerland (1)
	The Netherlands (3)
South Asia (170)	India (155)
	Indonesia (3)
	Malaysia (1)
	Pakistan (5)
	Philippines (2)
	Thailand (1)
	Turkey (3)
not assigned a world culture (6)	Algeria (1)
	Greece (1)
	Lebanon (1)
	Singapore (1)
	South Korea (1)
	Trinidad & Tobago (1)

### (c) Analysis

We used the statistical concept of model selection using maximum-likelihood estimation. Using this approach, a series of models are tested with each model representing a candidate hypothesis [45]. Akaike's information criterion (AIC) values were compared between different models. AIC values are calculated as −2 (ln (likelihood))+2K, where likelihood is the probability of the data given the model and K is the number of parameters in the model. A decrease in AIC corresponds to an increase in the fit of the model to the data or a reduction in the number of parameters. Thus, comparing AIC values allows you to pick the model that explains most of the variance in the data using the minimal number of explanatory terms. Moreover, the model selection approach deals to some extent with non-independence of explanatory terms (as was the case with income level and culture in this study), since models are penalized by the inclusion of additional terms. Thus, explanatory terms that are correlated with other terms in the model should only be retained if they independently improve the fit of the model.

In each case, we first considered a basic model, including only the constant and the residual variance. In subsequent models, we used a conservative approach of adding one explanatory term at a time to the basic model. Terms were considered to improve the fit of the model significantly if they lowered the AIC value by more than two units [45]. If more than one term received considerable support, we generated another model investigating both terms together. All statistical tests were performed using R v. 2.8.1 (www.r-project.org) and data conformed to the assumptions of the tests being used. Two-way interactions are presented only where significant at *p* < 0.05.

We asked whether donations in the dictator game were influenced by the presence of subtle social cues (eye images) and whether these effects, if they existed, were mediated by other demographic or cultural variables. Since the data could not be transformed to fit the assumptions of Gaussian models, we analysed the data using ordinal logistic regression. This approach allows ordered, categorical response terms to be modelled as a function of one or more independent variables. Dictator donations were split into eight ordered categories based on the most popular allocations chosen by dictators (see electronic supplementary material, table S1). Although dictators were free to choose any split of the $0.50 they desired, most individuals opted for donations that were multiples of $0.05 cents. Each category had a minimum of 10 independent data points (see electronic supplementary material, table S1). This categorical variable was set as the response term in series of ordinal logistic regression models. We compared a series of models, including age, culture, education level, gender, income level and image (eyes/flowers/control), as well as all two-way interactions, as explanatory terms.

For the analysis investigating the effect of culture on dictator donations, we restricted the dataset to cultures for which we had a minimum of 10 independent samples. This resulted in a restricted sample size of 360 dictators from four world cultures (Catholic Europe, English-speaking, Orthodox and South Asia, see table 1 for more details). Of these 360 dictators, three (0.008%) reported that they currently lived in a country different from their country of origin. Since it is unclear whether people who move countries are still the representative of their country of origin's cultural norms, we ran all models both including and excluding these data points. No qualitative differences were found and so we retained these individuals in the analysis presented here.

## 3. Results

**Figure 1.**
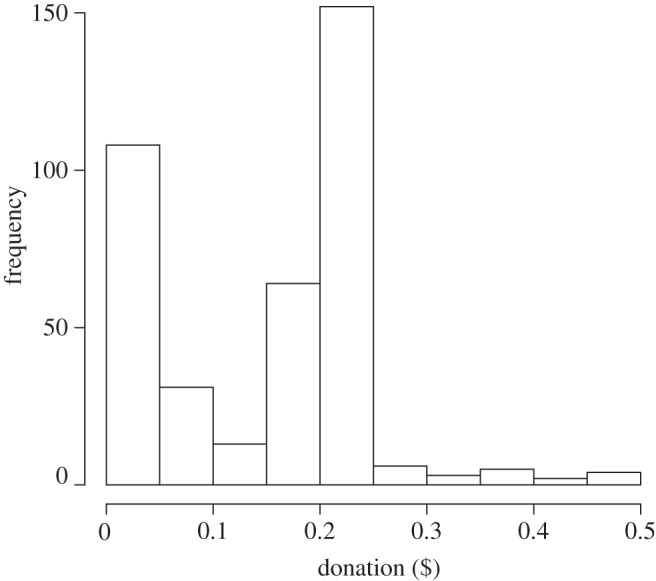
Histogram of dictator donations ($).

Although there was a range of dictator donations between $0.00 and $0.50, responses centred around being entirely selfish or giving half of the endowment to the receiver (figure 1). The mean amount donated to receivers by dictators in this one-shot dictator game was $0.17 ± 0.01, which corresponds to 34 per cent of the original endowment. The median donation was $0.2 (40%) and the mode was $0.25 (50%). Thus, most players split the endowment equally between themselves and the receiver, although less generous allocations were also common (figure 1).

**Figure 2.**
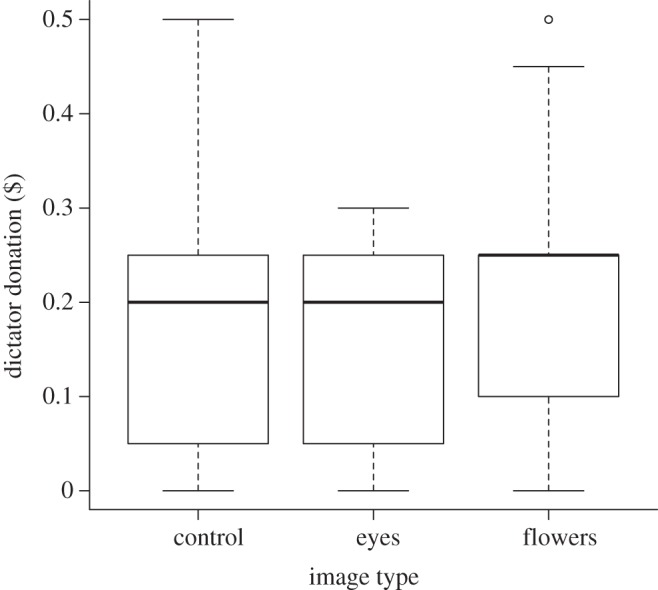
Boxplot of image type (eyes/flowers/control) against dictator donation ($). Boxplots display median values (solid lines) with inter-quartile ranges (upper and lower limits of the boxes). The maximum and minimum values of the data range are indicated by the dashed bars. Outliers are indicated with circles. Plots are generated from raw data and do not control for other significant terms affecting dictator donations.

**Figure 3.**
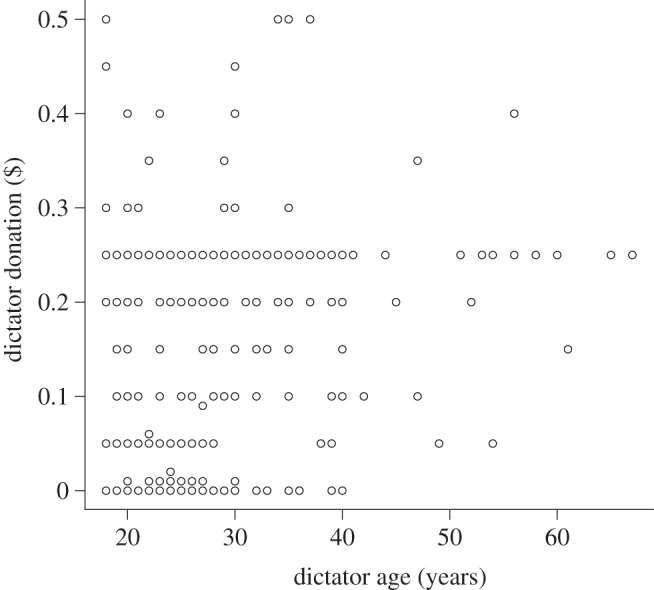
Scatterplot of dictator donations ($) according to self-reported age. Points represent raw data points and are not corrected for additional significant terms affecting dictator donations.

We investigated which terms affected dictator donations (table 2). Dictator donations varied according to the image type they were shown (figure 2). Dictator donations to receivers were highest when they were shown the image of flowers (mean donation = $0.19 ± 0.01) and lowest when they were shown eye images (mean donation = $0.15 ± 0.01). Donations were intermediate when dictators were presented with the black square image ($0.16 ± 0.01) or with no image ($0.17 ± 0.01). We found no significant difference in dictator donations when presented with the black square or when presented with no image (Wilcoxon test, *W* = 4679.5, *p* = 0.49) so these data were collated as a single ‘control’ treatment in the model. Comparison of effect sizes and s.e. for the different image types generated from the model revealed that there was no significant effect of eye images (effect size relative to ‘control’ image = −0.16 ± 0.22) on dictator donations, but that donations were significantly higher in response to flower images (effect size relative to ‘control’ image = 0.48 ± 0.23). Our model also showed that the average donation increased with the player's age (effect size = 0.03 ± 0.01; figure 3). Finally, we found a significant effect of culture on dictator donations (table 2 and see below). Including education level, gender and income level did not improve the fit of the model to the data. Similarly, no two-way interactions improved the fit of the model to the data.

**Table 2.** Table of candidate models (models including two-way interactions not shown) from ordinal logistic regression investigating variation in dictator donations ($) according to image type and demographic variables. Response term was an ordered categorical variable from one to eight denoting increasing dictator generosity (see electronic supplementary material, table S1 and methods for details). The best model is shown in bold. The basic model included the constant and the residual variance. All other models included the basic model plus the additional parameter(s) indicated. Lower AIC values indicate greater support for the candidate model. *Δ*AIC is the difference between that model and the model with the lowest AIC value. Models that generate AIC values within two units of each other are thought to receive equal support [45].

model	AIC	*Δ*AIC
1. basic	1367	15.4
2. age	1359.3	7.7
3. culture	1358.8	7.2
4. education	1366.8	15.2
5. gender	1370.7	19.1
6. image	1365.7	14.1
7. income	1368.4	16.8
8. age + culture	1354.9	3.3
9. **age + culture + image**	**1351**.**6**	**0**

**Figure 4.**
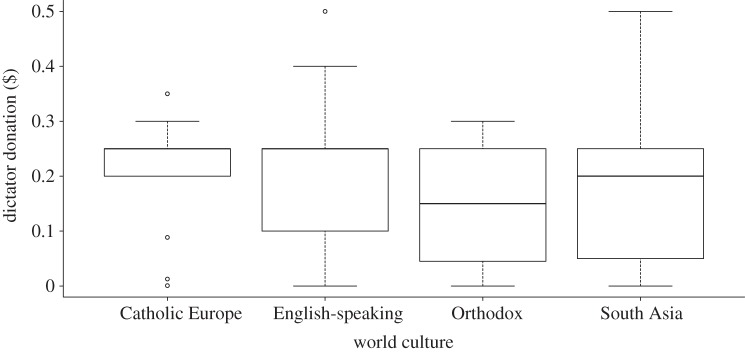
Boxplot of dictator donation ($) according to world culture. See figure 2 legend for description of how data are presented. Plots are generated from raw data and do not control for other significant terms affecting dictator donations.

Analysis on the dataset restricted to the four world cultures where we had at least 10 data points per culture showed that there was a significant effect of culture on dictator donations (figure 4 and table 3). The effects of image and age remained significant in this restricted dataset. There was no significant interaction between culture and image type, indicating that players from these four world cultures did not respond significantly differently to the different treatments.

**Table 3.** Table of candidate models (models including two-way interactions not shown) investigating variation in dictator donations ($) but based on a restricted subset of the data (*n* = 361) for which at least 10 data points per culture were available (see §2 and table 1 for details). Models were generated and compared as for table 2.

model	AIC	*Δ* AIC
1. basic	1270	17.8
2. age	1263.2	11
3. culture	1259.6	7.4
4. education	1270.1	17.9
5. gender	1273.3	21.1
6. image	1267	14.8
7. income	1271.3	19.1
8. age + culture	1255.3	3.1
9. **age + culture + image**	**1252**.**2**	**0**

## 4. Discussion

Previous studies have shown that minimal social cues increase contributions to cooperative endeavours [12–17]. Here, we found the opposite result: donations in a one-shot, anonymous dictator game were not significantly different when subjects were presented with eye images as opposed to a neutral image of a square or when there was no image. Surprisingly, donations were highest when subjects were shown images of flowers before making their donation. We suggest that a key difference between our study and previous studies that have demonstrated a positive effect of eye images on human cooperative behaviour is that subjects in our study most probably played under truly anonymous settings. Most AMT workers submit work from their own computers and may often be unobserved while they work. In contrast, several of the previous studies investigating the effect of eye images on cooperative behaviour have taken place in public settings [13,16], or in a laboratory with other players present [12,14,15]. Thus, rather than serving as an implicit cue that behaviour *is* being observed, eye images may simply serve to remind people that their behaviour *might* be observed by others. Since this cue is more likely to be valid in a public setting, eye images may be correspondingly more likely to induce cooperative behaviour in public rather than in private places. If, on the other hand, people believe their decisions are truly unobservable then minimal social cues such as eye images may not be expected to have demonstrable effects on cooperative behaviour.

The argument that human subjects know when their behaviour is truly anonymous is supported by a recent study using the Ultimatum Game [34]. This is a variant of the dictator game where receivers have veto power over the proposed division of the endowment [28,46]. If receivers reject the proposer's offer then neither player gets any money. Lamba & Mace [34] found that proposers made lower offers in the Ultimatum Game when they were told that their offer would be anonymous compared with when they were told that their offer would be made public. This effect of anonymity persisted regardless of whether proposers were seated alone in a room, or in the same room as fellow subjects (and therefore in the presence of potential observers). Similarly, another recent study using the dictator game placed dictators in a sound-proofed, darkened room before asking them to make their donation to receivers [21]. Under these conditions, the image of a human face on a computer screen did not increase donations significantly above those that were made when presented with a control image [21]. By the way of explanation, the authors suggested that in a dark, sound-proof room, dictators felt truly anonymous since behaviour may be less observable in real life under such conditions. These two studies suggest that cues of being watched might not be expected to affect cooperative behaviour in situations where players feel truly anonymous. In our study, AMT workers may feel truly anonymous since they interact via a computer and are not in the same room, or often even in the same country, as their interaction partner. Workers never expect to meet either the experimenter or their game partner in real life and the identity of all players was protected via the use of the artificial worker ID. Our study may therefore be one of the best approximations to a true double-blind version of the dictator game.

If these assumptions are valid, then two further patterns in our results appear to be striking and warrant explanation. First, donations in our game were on average $0.17, which represents 34 per cent of the initial $0.50 endowment. Data from a recent meta-analysis over 100 dictator game studies indicate that the mean donation is 28.3 per cent of the endowment [29]. Thus, our AMT dictators were significantly more generous than expected (Wilcoxon test performed on data supplied by C. Engel; *z* = −9.59, *p* < 0.001). Similarly, the meta-analytical approach showed that the modal donation to receivers is 0 per cent, and around 17 per cent of dictators opt for an equal division of the endowment [29]. By contrast, in our study, just 11.6 per cent of dictators in our study kept the entire endowment and gave nothing to the receiver. Instead, the modal donation was 50 per cent of the endowment, with 37.5 per cent of dictators opting for this division. Thus, our dictators seemed to be more generous than expected, particularly when compared with dictators in double-blind settings, where mean offers may be as low as 10 per cent of the endowment [28,47].

It could be argued that the discrepancies between our results and results reported from other studies is an artefact of our relatively low stake size of $0.50 (the mean stake size used across all previous dictator game studies was $21.8 [29]). However, this would seem to be an unlikely explanation. While Engel [29] demonstrated a very small negative effect of stake size on donations in games where stakes were manipulated during the game, comparisons across studies where the stake size is invariant (as in our study) typically reveal no consistent effect of stake size on average donations in dictator games [28,29]. We have also shown that increasing the stakes to $5 and $10 does not significantly affect dictator donations in AMT dictator games (N. J. Raihani, R. Mace & S. Lamba 2012, unpublished data). Furthermore, previous studies have validated the AMT approach using small stake sizes in both a prisoner's dilemma game [37] and a public goods game. In both these studies, the stakes presented to AMT workers were an order of magnitude smaller than the stakes used in the physical laboratory setting, but the data revealed no significant difference subject behaviour between the two settings [37,43]. An alternative explanation for the relatively high dictator donations in our study is that AMT dictators felt as though both individuals had worked to earn a reward. Previous work has shown that dictator allocations to receivers are influenced by the extent to which dictators feel entitled to the endowment. In games where dictators have to work to earn the endowment, they typically give little to receivers [48], while in games where receivers work to earn the endowment, donations are typically higher [49,50]. In games where both players work to earn the endowment, dictator allocations to receivers tend to reflect the relative effort of each player, with dictators allocating more to receivers if receivers are responsible for earning more of the endowment [51]. In the AMT setting, dictators may have felt as though receivers were entitled to some portion of the endowment since both players were required to fill out a questionnaire prior to the allocation decision made by the dictator. The perception that receivers were entitled to some of the endowment may have been particularly pronounced since players on AMT are referred to and consider themselves as ‘workers’, reflecting the fact that they provide labour in return for financial recompense. Such subtle framing effects have been shown to influence dictator behaviour in previous studies [37,52]. Finally, it is possible that the AMT dictators were more generous than dictators in other studies because they represent a more diverse slice of society. In particular, undergraduate students—the most frequently sampled demographic—tend to make less generous allocations than non-students in the dictator game [29]. While we do not know what proportion of our AMT dictators were undergraduate students, the self-reported information about education level confirms that at least 122 players (65% of our sample) are not a current undergraduate. This large body of non-students may partly explain why our dictators appeared to make generous donations, when compared with similar studies.

A second striking result of this study is that donations were significantly higher when subjects were shown images of flowers than when shown images of eyes or in a control treatment. This is precisely opposite to the result we initially predicted based on previous studies [12–17]. While we have proposed an explanation as to why eye images did not result in increased cooperative behaviour relative to controls, it remains to be explained why dictators should make higher donations after seeing images of flowers. We suggest that the effect of flowers on dictators' behaviour may have arisen through the established associations between viewing nature and positive emotional state. Previous work has shown that access to nature can have a profound effect on people's health, wellbeing and responses to stressful situations [53]. Importantly, these positive effects can arise even through indirect interactions with nature, such as viewing a scene or an image of nature [54]. For example, in a study measuring patient outcomes following surgery, Ulrich [55] found that patients with views of nature recovered faster, required fewer painkillers and spent less time in hospital compared with matched patients whose beds overlooked urban views. Further work has shown that artificial scenes of nature can also have profound effects on psychological state. Specifically, viewing a scene of nature as opposed to an urban scene can promote feelings of pleasure and enhance attention, while at the same time decreasing negative emotions such as anger or anxiety [56]. Positive emotional state, in turn, has been linked to increases in sociable and cooperative behaviour [57]. Thus, based on this previous body of research, we suggest that flower images might have induced positive emotional states in our dictator game players, resulting in the tendency for dictators to donate more to receivers when presented with flower images.

We also found that dictator donations were significantly and positively associated with dictator age. The positive effect of age on giving in dictator games has been demonstrated before, both in children [58,59] and adults [60–62] (but see [29]). Among children, it is argued that age has a bearing on cooperative behaviour since social norms of cooperation have a learned component rather than being exclusively genetically determined [63]. In addition, children's sensitivity to the opinions of others might increase with age, since an understanding of how one's behaviour might influence the opinions and beliefs of others rests on sophisticated cognitive processes that very young children (under 3 years old) are thought to lack [64]. It is less clear why altruistic behaviour should increase with age in adults. One hypothesis is that disposable income may increase more with age, thereby predisposing older subjects to more charitable behaviour. Although we controlled for income in our study—and found no significant effect of income on donations in the dictator game—we do not have a clear measure of disposable income for our subjects. It is plausible, however, that disposable income increases with age, particularly if this coincides with the period when any children become independent [65]. Future approaches could ask subjects to indicate how many dependent children they have, as well as to estimate monthly disposable income (rather than income per se) to provide a more rigorous investigation of why cooperative behaviour increases with age.

We found no effects of gender on dictator donations. While some previous studies have indicated that females are generally more altruistic than males in dictator games [66,67], others have found the opposite result [15], or report no effect of gender [12,68,69]. The meta-analysis approach revealed a significant effect of gender on dictator game giving: women both give more and receive more in dictator games [29]. Despite this result, the overall picture of gender effects on donations in dictator games remains quite mixed [28], and the precise conditions under which females are expected to donate more than males are not currently well understood.

One of our stated aims was to use the AMT framework to explore the potential effects of cross-cultural variation in dictator game giving. In particular, we were interested in whether we would find cross-cultural variation in sensitivity to minimal social cues of being watched. Cultural differences have been associated with differences in cooperative behaviour both in small-scale [23,24,70,71] and large-scale societies [25,26,72]. While we found a significant effect of culture on dictator donations, we did not find a significant interaction between culture and image type on dictator donations. This indicates that, for the four world cultures where we had a sufficient sample size to address this question, there was no systematic variation in how players responded to different image types. It is currently unclear why players from different world cultures should make different donations in the dictator game, although cross-cultural variation in prosocial behaviour has been shown before [23,24,71,72]. In small-scale societies, cross-cultural variation in market integration (the percentage of calories consumed that are bought as opposed to hunted or grown) significantly and positively predicts dictator game donations [71]. It has been argued that preferences for fairness are favoured in increasingly industrialized societies because such preferences increase the efficiency of exchanges, and thereby facilitate the most productive use of unevenly distributed skills, resources and knowledge [71]. However, cross-cultural differences in cooperative behaviour also exist in large-scale societies, which cannot be attributed to differences in market integration [72,73]. Instead, variation in behaviour may arise because these cultures have different historical and cultural values [44,73]. Nevertheless, the precise reason why large-scale differences in values across cultures might affect cooperative behaviour remains obscure.

To summarize, we used an online labour market to investigate whether subtle cues of being watched would affect donations in a double-blind dictator game. Contrary to our initial predictions, we found that dictators were more generous when shown images of flowers than when they were shown eye images or other control images. This may be because players felt truly anonymous in the context of our experimental setting, and because flower images produced positive emotional effects and thereby increased donations. Our findings highlight the need to explore more rigorously the conditions under which subtle cues of being watched enhance cooperative behaviour.
